# Surgical treatment of esophageal atresia with lower tracheoesophageal fistula in an extremely preterm infant (510 g, 25 + 5 weeks): a case report

**DOI:** 10.1186/s13256-021-02951-x

**Published:** 2021-07-12

**Authors:** Xiaoyan Feng, Ulrich Thomé, Holger Stepan, Martin Lacher, Richard Wagner

**Affiliations:** 1 Department of Pediatric Surgery, University Hospital Leipzig, University of Leipzig, Liebigstrasse 20a, 04103 Leipzig, Germany; 2 Department of Neonatology, University Hospital Leipzig, University of Leipzig, Leipzig, Germany; 3 Department of Obstetrics, University Hospital Leipzig, University of Leipzig, Leipzig, Germany

**Keywords:** Tracheoesophageal fistula, Extremely low birth weight, Extremely preterm, Thoracoscopic repair, Case report

## Abstract

**Background:**

The surgical management of esophageal atresia in extreme-low-birth-weight infants (< 1000 g) is challenging. We report on an extreme-low-birth-weight infant who was extremely preterm (510 g, 25 + 5 weeks) and of prenatally unknown Gross type C esophageal atresia.

**Case presentation:**

After resuscitation and intubation, the tracheoesophageal fistula was closed on the first day of life in the neonatal intensive care unit via an extrapleural approach using a titanium clip. On the sixth day of life, the Caucasian child was extubated. To minimize the operative trauma in the initial neonatal period, we prolonged gastrostomy placement until the 22nd day of life (weight 725 g). At the age of 3 months (weight 2510 g), thoracoscopic esophageal anastomosis was performed. The postoperative course was unremarkable. During the further clinical course, eight esophageal dilations were necessary. Currently, the patient swallows without difficulties at the age of 4 years and thrives well [15 kg (Percentile 28); 100 cm (Percentile 24)].

**Conclusions:**

Our case shows that minimized postnatal surgical trauma with primary tracheoesophageal fistula closure at the bedside, delayed gastrostomy, and minimally invasive esophageal repair after substantial weight gain (> 2.5 kg) is a good strategy for esophageal atresia/tracheoesophageal fistula in extreme-low-birth-weight infants.

**Supplementary Information:**

The online version contains supplementary material available at https://doi.org/10.1186/s13256-021-02951-x.

## Background

The percentage of esophageal atresia (EA) with tracheoesophageal fistula (TEF) in extreme-low-birth-weight (ELBW) infants (< 1000 g) is low (3%) [1]. For such cases, mortality can be very high (50–100%) [1, 2]. The surgical strategy for these children is controversial. Some argue that the anastomotic result after primary EA repair is favorable compared with staged repair [3]. In contrast, others advocate that primary fistula ligation followed by gastrostomy and delayed esophageal anastomosis achieved better outcomes and lowered the rate of anastomotic complications in ELBW infants [1, 4]. Here, we describe a case of staged operation in an ELBW and extremely preterm infant (510 g, 25 + 5 weeks) who was born with prenatally unknown EA/TEF (Gross type C).

## Case presentation

A 510 g Caucasian female infant without prenatally diagnosed anomalies was delivered to a previously healthy 37-year-old mother (gravida 7, para 4, ab 2) at 25.7 weeks gestation via cesarean section after pathological findings on cardiotocography and growth retardation. Apgar scores were 3, 6, and 7. The patient was intubated immediately after birth, and broad-spectrum antibiotics (ampicillin + cefotaxime) were started. EA/TEF (Gross type C) was diagnosed via chest x-ray after a nasogastric tube was unable to pass the esophagus. A Replogle tube was placed in the upper pouch with continuous suction, and an umbilical vein catheter was established. Cardiac and renal ultrasound were normal; no additional malformations were diagnosed. The three older siblings of the patient do not have any congenital anomalies and are all healthy.

**Fig. 1:**
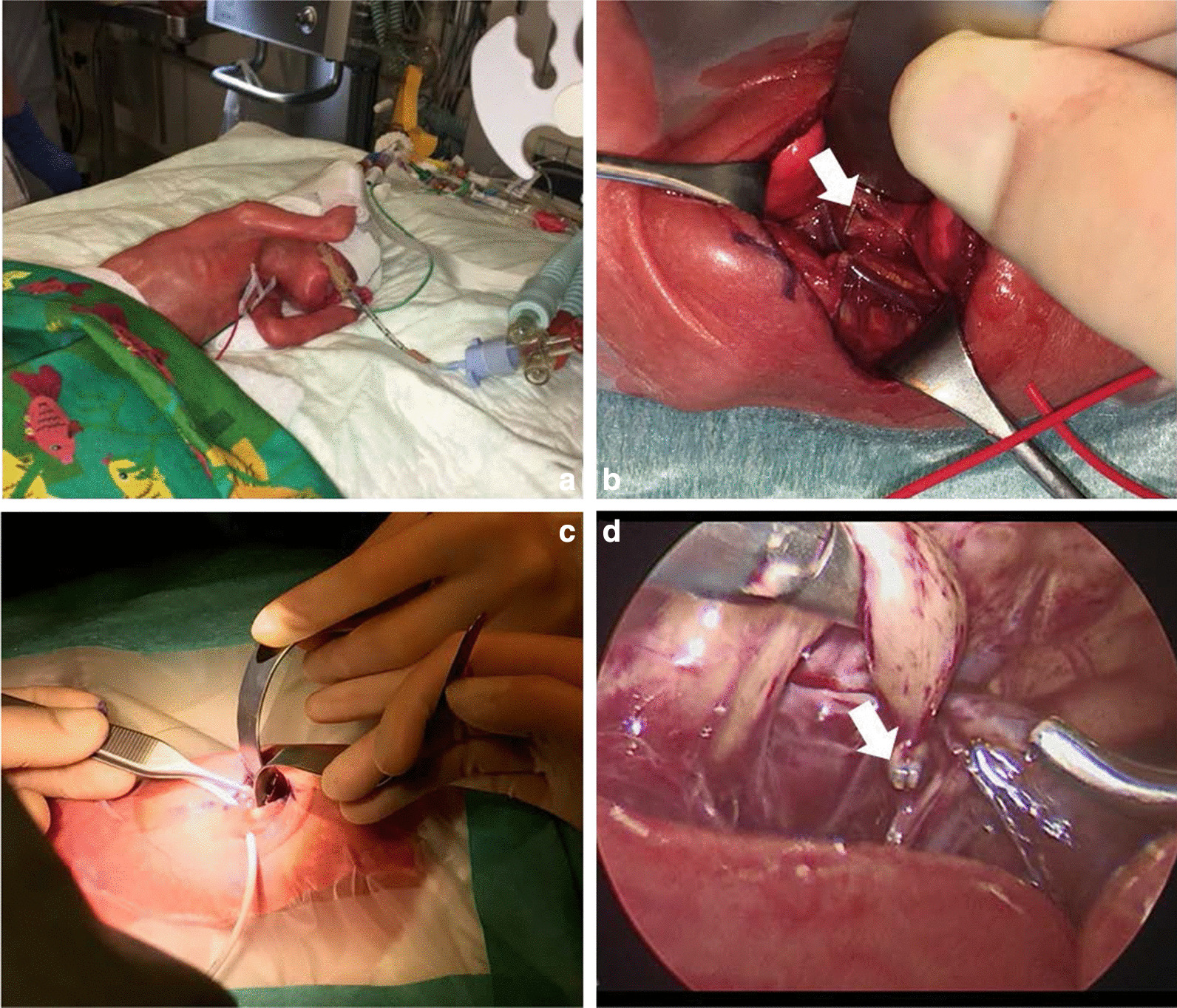
The surgical procedure of the patient. **A** The patient was intubated immediately after birth; open tracheoesophageal fistula closure was performed in the neonatal intensive care unit. **B** Titanium clip was used to ligate the tracheoesophageal fistula. **C** On 22nd DOL, gastrostomy was created. **D** Three months after birth, esophageal anastomosis was performed via thoracoscopy. White arrow in **B** and **D** indicates location of titanium clip

On the first day of life (DOL) (10 hours after birth) open TEF clipping was performed via an extrapleural approach in the incubator at the neonatal intensive care unit using a titanium clip (operation time 36 minutes). To reduce the operative trauma in the initial neonatal period and enable extubation after 6 days, we decided to postpone the placement of a gastrostomy. Therefore, we applied total parenteral nutrition (TPN), which resulted in a constant elevation of direct bilirubin (maximum of 200 mg/dl). At 22 DOL (weight 725 g), we created an open gastrostomy (operation time 58 minutes). After steady weight gain (2510 g), we performed a thoracoscopic primary esophageal anastomosis (operation time 93 minutes) at 3 months of age, without tension and without any perioperative adverse events (Fig. 1). The patient was weaned from mechanical ventilation after 4 days. A transanastomotic tube was left in place until an upper contrast swallow study was performed 7 days after surgery. After 14 days, the patient was completely on oral feeds.

Upper endoscopy including balloon dilatations started routinely after 1.5 months postsurgery. There was no event of complete stricture. However, we performed eight dilatations during the first year of life until the anastomotic stenosis resolved. Although the patient experienced one to three respiratory infections per year, there were no events of aspiration pneumonia or sepsis. Currently, the patient swallows effortlessly at the age of 4 years and thrives well [15 kg (Percentile 28); 100 cm (Percentile 24)].

## Discussion

The optimal surgical treatment for EA/TEF in ELBW infants (< 1000 g) remains controversial (Additional file 1). To date, there are eight case reports and two comparative studies that include children with less than 445 g and 23 weeks gestation. In 1992, Schaarschmidt *et al.* reported staged repair in an infant born with 445 g at of 36 weeks gestation that survived [5]. Gastrostomy on second DOL was followed by open TEF ligation and primary esophageal anastomosis after 29 days. In 2016, Zani *et al.* reported a case with 540 g of 24 weeks gestational age with initially diagnosed pure EA (based on bronchoscopy findings). [1] The patient underwent gastrostomy placement after 61 days. On the 126th DOL, the presence of a proximal fistula was noticed during open surgery, and TEF ligation as well as esophageal anastomosis was achieved. The authors compared the outcomes of primary versus staged repair in a total of eight patients. They concluded that initial TEF ligation followed by delayed esophageal repair is better than primary anastomosis in ELBW infants. Another comparative study showed that 20% (1/5) of ELBW patients with EA/TEF survived after primary repair, while 100% (2/2) pure EA and 100% (2/2) EA/TEF survived after a staged operation [6]. The authors argued that a minimal initial operative trauma is lifesaving for such ELBW patients and therefore recommend delayed esophageal anastomosis.

Likewise, there are some publications that report successful primary repair in ELBW infants [7, 8]. None of the surviving patients had severe associated anomalies (for example, major cardiac anomalies, renal deformity, chromosomal abnormalities), and birth weight in all cases was > 700 g. Therefore, patient selection may be a crucial factor for the success of primary repair.

Malnutrition and growth failure are frequent problems in ELBW patients with EA/TEF. Therefore, parenteral nutrition must be initiated soon after delivery to prevent postnatal failure to thrive. Our patient showed total parenteral-nutrition-dependent weight gain from 510 g to 725 g until the 22nd DOL. One major complication of total parenteral nutrition is parenteral-nutrition-associated liver disease (incidence 25–60%) [9, 10]. Nevertheless, we tolerated a temporary elevation of liver enzymes and bilirubin levels until we performed the open gastrostomy on the 22nd DOL. Thereafter, we started full enteral feeds, which resulted in an increase of weight from 725 g to 2510 g (90th DOL).

Thoracoscopic repair (TR) of EA/TEF is an ongoing controversy. Dingemann *et al.* suggested that neonates with a short-gap EA that are cardiopulmonary stable and weigh more than 2000 g at birth with less than one major associated malformation are good candidates for TR [11]. Others demonstrated that TR can be performed safely and effectively by experienced surgeons, and patients with even lower birth weights (< 1800 g). However, major coexisting anomalies and hemodynamically unstable patients shall be excluded from TR [12, 13]. One meta-analysis compared the clinical outcomes between open repair and TR for EA/TEF involving 447 studies. It showed that TR can reduce the length of hospital stay, time to first oral feeds, and long-term musculoskeletal morbidity. In contrast, operation times were longer in the TR group. The rates of leakage, stricture, pulmonary complications, and the need of fundoplication due to gastroesophageal reflux disease were similar in the open and TR groups [14].

Reports on delayed TR for EA/TEF in ELBW infants are rare. Margain *et al.* report on three cases with birth weights and gestational ages of 490, 790, and 800 g and 25, 26, and 27 weeks, respectively [15]. They were primary treated with lower esophageal banding, followed by delayed thoracoscopic esophageal repair and dissection of the fistula. Two patients survived with uneventful courses, and delayed TR was performed at 70 and 80 days DOL and 2100 and 2200 g, respectively. These data match our patient, who was born at 25 weeks gestation with a birth weight of 510 g; delayed thoracoscopic anastomosis was performed after 3 months at 2510 g.

## Conclusions

Extremely preterm and low-birth-weight infants of EA/TEF can benefit from a staged repair with primary open ligation of the fistula followed by gastrostomy and delayed thoracoscopic anastomosis.

## Supplementary Information

**Additional file 1. **Summary of surgical treatment for EA/TEF in ELBW infants (< 1000g)

## Data Availability

Not applicable.

## Abbreviations

EA: Esophageal atresia
TEF: Tracheoesophageal fistula
ELBW: Extreme low birth weight
DOL: Day of life
TR: Thoracoscopic repair

## References

[1] Zani A, Wolinska J, Cobellis G, Chiu PPL, Pierro A. Outcome of esophageal atresia/tracheoesophageal fistula in extremely low birth weight neonates (<1000 grams). Pediatr Surg Int. 2016;32:83–8. 10.1007/s00383-015-3816-7 26519042

[2] Schmidt A, Obermayr F, Lieber J, Gille C, Fideler F, Fuchs J. Outcome of primary repair in extremely and very low-birth-weight infants with esophageal atresia/distal tracheoesophageal fistula. J Pediatr Surg. 2017;52:1567–70. 10.1016/j.jpedsurg.2017.05.011 28554817

[3] Dingemann C, Brendel J, Wenskus J, Pirr S, Schukfeh N, Ure B, *et al*. Low gestational age is associated with less anastomotic complications after open primary repair of esophageal atresia with tracheoesophageal fistula. BMC Pediatr. 2020;20:267. 10.1186/s12887-020-02170-1 PMC7268419 32493241

[4] Petrosyan M, Estrada J, Hunter C, Woo R, Stein J, Ford HR, *et al*. Esophageal atresia/tracheoesophageal fistula in very low-birth-weight neonates: improved outcomes with staged repair. J Pediatr Surg. 2009;44:2278–81. 10.1016/j.jpedsurg.2009.07.047 20006009

[5] Schaarschmidt K, Willital GH, Jorch G, Kerremanns J. Delayed primary reconstruction of an esophageal atresia with distal esophagotracheal fistula in an infant weighing less than 500 g. J Pediatr Surg. 1992;27:1529–31. 10.1016/0022-3468(92)90495-s 1469562

[6] Hannon EJ, Billington J, Kiely EM, Pierro A, Spitz L, Cross K, *et al*. Oesophageal atresia is correctable and survivable in infants less than 1 kg. Pediatr Surg Int. 2016;32:571–6. 10.1007/s00383-015-3851-4 PMC4870296 27090660

[7] Driver CP, Bruce J. Primary reconstruction of esophageal atresia with distal tracheoesophageal fistula in a 740-g infant. Pediatr Surg Int. 1997;32:1488–9. 10.1016/s0022-3468(97)90571-6 9349778

[8] Seitz G, Warmann SW, Schaefer J, Poets CF, Fuchs J. Primary repair of esophageal atresia in extremely low birth weight infants: a single-center experience and review of the literature. Biol Neonate. 2006;90:247–51. 10.1159/000094037 16785729

[9] Calkins KL, Venick RS, Devaskar SU. Complications associated with parenteral nutrition in the neonate. Clin Perinatol. 2014;41:331–45. 10.1016/j.clp.2014.02.006 PMC5384841 24873836

[10] Nandivada P, Fell GL, Gura KM, Puder M. Lipid emulsions in the treatment and prevention of parenteral nutrition-associated liver disease in infants and children. Am J Clin Nutr. 2016;103:629S-S634. 10.3945/ajcn.114.103986 PMC4733251 26791189

[11] Dingemann C, Zoeller C, Ure B. Thoracoscopic repair of oesophageal atresia: results of a selective approach. Eur J Pediatr Surg. 2013;23:14–8. 10.1055/s-0032-1326953 23093437

[12] Okuyama H, Saka R, Takama Y, Nomura M, Ueno T, Tazuke Y. Thoracoscopic repair of esophageal atresia. Surg Today. 2019. 10.1007/s00595-019-01884-9. 31612332

[13] Holcomb GW. Thoracoscopic surgery for esophageal atresia. Pediatr Surg Int. 2017;33:475–81. 10.1007/s00383-016-4049-0 28064362

[14] Wu Y, Kuang H, Lv T, Wu C. Comparison of clinical outcomes between open and thoracoscopic repair for esophageal atresia with tracheoesophageal fistula: a systematic review and meta-analysis. Pediatr Surg Int. 2017;33:1147–57. 10.1007/s00383-017-4153-9 28914345

[15] Margain L, Perez-Etchepare E, Varlet F, Lopez M. Lower esophageal banding in extremely low birth weight infants with esophageal atresia and tracheoesophageal fistula is a life saving practice followed by a successful delayed primary thoracoscopy reconstruction. J Pediatr Surg. 2015;50:489–92. 10.1016/j.jpedsurg.2014.10.055 25746714

