# Supplementary material for: Surgical treatment of esophageal atresia with lower tracheoesophageal fistula in an extremely preterm infant (510 g, 25 + 5 weeks): a case report
Source: J Med Case Rep. 2021 Jul 12;15:361. doi: 10.1186/s13256-021-02951-x (PMC8273969; doi:10.1186/s13256-021-02951-x)
Supplement: Supplementary file 1 — Additional file 1. Summary of surgical treatment for EA/TEF in ELBW infants (< 1000g) [file 13256_2021_2951_MOESM1_ESM.docx]

**Supplement:** Summary of surgical treatment for EA/TEF in ELBW infants (< 1000g)
